# ADAM17 Activity and IL-6 Trans-Signaling in Inflammation and Cancer

**DOI:** 10.3390/cancers11111736

**Published:** 2019-11-05

**Authors:** Neele Schumacher, Stefan Rose-John

**Affiliations:** Biochemical Institute, University of Kiel, 24118 Kiel, Germany; nschumacher@biochem.uni-kiel.de

**Keywords:** ADAM17, interleukin-6, trans-signaling, epidermal growth factor receptor (EGF-R), shedding, metalloprotease, tumor necrosis factor alpha (TNFα), inflammation associated cancer, colon cancer, lung cancer

## Abstract

All ligands of the epidermal growth factor receptor (EGF-R) are transmembrane proteins, which need to be proteolytically cleaved in order to be systemically active. The major protease responsible for this cleavage is the membrane metalloprotease ADAM17, which also has been implicated in cleavage of TNFα and interleukin-6 (IL-6) receptor. It has been recently shown that in the absence of ADAM17, the main protease for EGF-R ligand processing, colon cancer formation is largely abrogated. Intriguingly, colon cancer formation depends on EGF-R activity on myeloid cells rather than on intestinal epithelial cells. A major activity of EGF-R on myeloid cells is the stimulation of IL-6 synthesis. Subsequently, IL-6 together with the ADAM17 shed soluble IL-6 receptor acts on intestinal epithelial cells via IL-6 trans-signaling to induce colon cancer formation, which can be blocked by the inhibitor of IL-6 trans-signaling, sgp130Fc. Blockade of IL-6 trans-signaling therefore offers a new therapeutic window downstream of the EGF-R for the treatment of colon cancer and possibly of other EGF-R related neoplastic diseases.

## 1. Introduction

**Figure 1:**
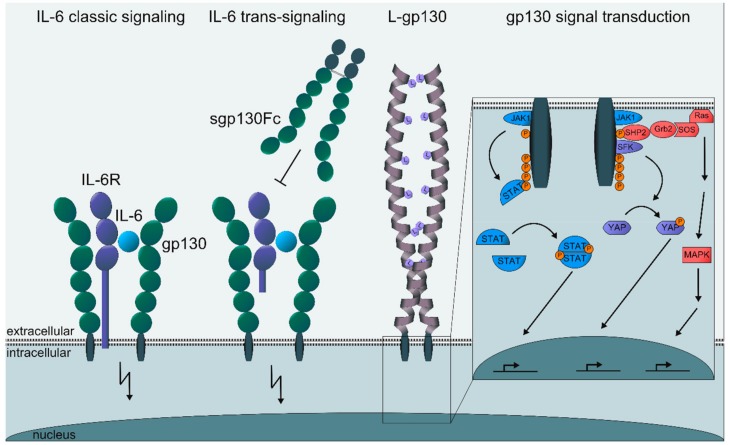
Modes of IL-6 signaling. IL-6 can signal via the membrane-bound IL-6R, which, however, is only expressed on some cell types. Alternatively, IL-6 can bind to the soluble IL-6R (sIL-6R) and the complex of IL-6/sIL-6R can stimulate virtually all cells in the body since gp130 is ubiquitously expressed. In an artificial model of cell-autonomous gp130 stimulation, the extracellular portion of gp130 has been exchanged for a leucine zipper, leading to constitutive gp130 signaling in the absence of any ligand [6]. The insert shows the main intracellular signaling pathways stimulated by gp130 activation.

IL-6 is a four-helical cytokine with pleiotropic activities, which is synthesized by many cell types upon appropriate stimulation and which can act on many cell types during several disease states such as inflammation and cancer [1]. IL-6 exerts its function through binding to the alpha-receptor Interleukin-6 receptor (IL-6R). The complex of IL-6 and IL-6R binds to the beta-receptor glycoprotein 130 (gp130) and induces dimerization of gp130 [2]. This dimerized gp130 leads to an activation of the tyrosine kinase Janus kinase 1 (JAK1), which is constitutively associated with the cytoplasmic portion of gp130. JAK1 phosphorylates tyrosine residues within the cytoplasmic portion of gp130. This leads to the recruitment of the adapter protein and phosphatase SHP2 that initiates MAP kinase and PI3 kinase signaling [3]. Furthermore, phosphorylated tyrosine residues recruit the cytoplasmic transcription factors STAT1 and STAT3, which thereupon become phosphorylated, dimerize, and translocate into the nucleus, where they bind to DNA and stimulate the transcription of gp130 target genes [3]. One of the earliest gp130 target genes is the gene coding for SOCS3. The SOCS3 protein is recruited to the membrane proximal tyrosine residue within the gp130 cytoplasmic tail where it inhibits the activity of JAK1. Therefore, SOCS3 is a negative feedback inhibitor of gp130 signaling [3]. Recently, it was shown that gp130 activation leads to the phosphorylation and activation of the YAP pathway (Figure 1). This pathway was shown to be important in the development of colon cancer [4,5].

While gp130 is expressed on all cells in the body, IL-6R is only expressed on some cells including hepatocytes, some leukocytes and some epithelial cells. Since IL-6 shows only measurable binding to the IL-6R but not to gp130, it follows that IL-6 can only act on cells, which express the IL-6R [2,7]. Interestingly, the membrane-bound IL-6R can be cleaved from the cell membrane by A disintegrin and metalloproteinases (ADAMs), resulting in a soluble form of the receptor [8,9,10]. The soluble IL-6R (sIL-6R) can still bind its ligand IL-6 and the complex of IL-6 and sIL-6R can bind to gp130 and induce dimerization of signaling. This mode of signaling has been called IL-6 trans-signaling [11]. Since gp130 is ubiquitously expressed, IL-6 trans-signaling can lead to the stimulation of virtually every cell in the body (Figure 1) [12]. However, there are few proteases described that can cleave membrane-bound IL-6R thus enabling IL-6 trans-signaling. Besides, the closely related ADAM proteases ADAM10 and ADAM17, meprin metalloproteases were reported to release the soluble form of the IL-6R [13,14,15,16].

Moreover, since on most cells gp130 is expressed at higher levels than IL-6R, stimulation by IL-6 leads to the stimulation of only some gp130 molecules whereas stimulation with the IL-6/sIL-6R complex leads to the activation and stimulation of all gp130 proteins resulting in a higher signal amplitude as a consequence of IL-6 trans-signaling as compared with IL-6 signaling via the membrane-bound IL-6R. IL-6 upon binding to the IL-6R is rapidly internalized in contrast to the IL-6/sIL-6R complex, which is internalized only with low efficacy [17]. This resulted in significantly longer stimulation mediated by the IL-6/sIL-6R complex as compared to IL-6 alone [17]. This property of IL-6 trans-signaling might be important for several cellular or tissue responses, which can only be induced by IL-6 trans-signaling but not by IL-6 classic signaling via the membrane-bound IL-6R [18].

Importantly, IL-6 trans-signaling can be specifically blocked by the extracellular portion of gp130 fused to the Fc-portion of a human IgG1 antibody (sgp130Fc) [1,19]. This blockade of IL-6 trans-signaling does not affect activities of IL-6 via the membrane-bound IL-6R. In transgenic mice overexpressing the sgp130Fc, IL-6 trans-signaling is completely blocked. These mice have been widely used to define the role of IL-6 classic signaling and IL-6 trans-signaling in many mouse models of human diseases (Figure 1) [1,12,20].

In healthy individuals, IL-6 levels are in the range between 1 and 5 pg/mL, but they can increase by many orders of magnitude during inflammatory states or upon infection. Plasma IL-6 levels of up to several µg/mL have been reported in patients with septic shock [21]. Interestingly, the level of sIL-6R in the blood of healthy individuals is in the range of 40–80 ng/mL, more than 1000 times higher than IL-6 [20]. A soluble form of gp130, which is generated by differential splicing [22] is found in the plasma of healthy individuals at approximately 400 ng/mL [20]. IL-6 binds to the IL-6R with an affinity of about 1 nM, whereas the complex of IL-6 and sIL-6R binds to gp130 with an affinity of 10 pM. Therefore, IL-6 released into the circulation will bind to sIL-6R and thereafter be trapped by sgp130. Thus, sIL-6R and sgp130 form a buffer for IL-6 [23]. The important function of the IL-6 buffer was underlined by studies on the coding single nucleotide polymorphism (SNP; rs2228145) in the human IL-6R that results in the alteration of Asp358 into Ala358. These amino acid residues are in close proximity of the proteolytic cleavage site of the IL-6R [16]. Interestingly, the IL-6R with Ala358 is a better protease substrate and is shed more efficiently leading to significantly increased plasma levels of sIL-6R [24]. Patients with the Ala358 variant of the IL-6R not only show about 2-fold elevated levels of sIL-6R [25], but they are also protected from inflammatory diseases such as congestive heart disease, abdominal aneurism, and rheumatoid arthritis [26,27,28]. Moreover, patients with the rs2228145 SNP showed a significant lower risk of Crohn’s disease and ulcerative colitis as compared to control individuals [29]. Furthermore, growth rates of abdominal aortic aneurisms in patients were smaller with the Ala358 variant of the IL-6R [30]. These results can be explained by a higher capacity of the IL-6 buffer in the blood, which is caused by the higher circulating sIL-6R levels [20,23].

IL-6 is synthesized and secreted by many cell types and the cytokine also acts on many cell types. IL-6 transgenic animals and IL-6 knock-out animals have been generated to define the major activities of this cytokine [31,32,33,34,35,36,37]. In order to explore cell-specific activities of IL-6, we generated a constitutively active gp130 construct. To this end, we replaced the entire extracellular portion of gp130 by a leucine zipper of the c-jun oncogene. Such an arrangement had been used to generate constitutively active cytokine receptor complexes [38]. Transfection of various cell types with the leucine zipper dimerized gp130 molecule, which we called L-gp130, was shown to lead to phosphorylation of STAT3 and to long-term growth of IL-6 dependent cells. Moreover, mouse embryonic stem cells transfected with the L-gp130 cDNA construct remained undifferentiated even in the absence of exogenously supplied LIF, indicating that cell autonomous gp130 activation had been achieved [6]. Interestingly, these data also supported the view that sustained gp130 signaling could not be completely switched off by the induction of the negative feed-back inhibitor protein SOCS3 (Figure 1) [6]. Recently, we inserted the L-gp130 cDNA construct into the ROSA26 locus of the mouse. In these mice, by breeding to an appropriate cre transgenic mouse, the L-gp130 molecule can be switched on constitutively in a cell autonomous manner. Activating gp130 in B-cells or in the entire hematopoietic system resulted in B-cell lymphomas and plasma cell disorders with full penetrance [39]. These mice will be useful in the future to study many more cell-specific activities of IL-6 without confounding activities of other cells or tissues.

ADAM17 is a membrane-bound metalloprotease, which was originally identified as the protease responsible for cleavage of the membrane-bound cytokine TNFα [40,41]. Upon gene deletion of ADAM17, it turned out that this protease is essential for life and it is also responsible for cleavage of ligands of the EGF-R [42]. In the following years, it was found that ADAM17 is also responsible for cleavage of the membrane-bound IL-6R [43] and for around 80 additional transmembrane protein substrates [44,45]. Conditional deletion of ADAM17 [46] and analysis of hypomorphic ADAM17^ex/ex^ mice [47] revealed important roles of the protease in immune regulation and homeostasis of many different tissues [44], including the central nervous system [45]. ADAM17 is expressed in most types of tissues but the cell surface activity of ADAM17 seems to be tightly regulated [44]. It turned out that intracellular trafficking of ADAM17 through the Golgi to the plasma membrane requires the presence of iRhoms, which are members of the intramembrane rhomboid proteases and which have lost protease activity in the course of evolution [48]. In the absence of iRhom1 or iRhom2, ADAM17 mRNA is expressed but the protein does not reach the cell surface [49,50]. Moreover, an additional protein, FRMD8 or iTAP, which binds to iRhom2 is involved in the activation of the ADAM17 protease via phosphorylation [51,52]. An additional mechanism of ADAM17 activation is the cell surface exposure of phosphatidylserine, which binds to a cationic phosphatidylserine-binding motif within the membrane-proximal domain of ADAM17 [53]. This mechanism explains the profound activation of ADAM17 by all inducers of apoptosis analyzed thus far [54].

**Figure 2:**
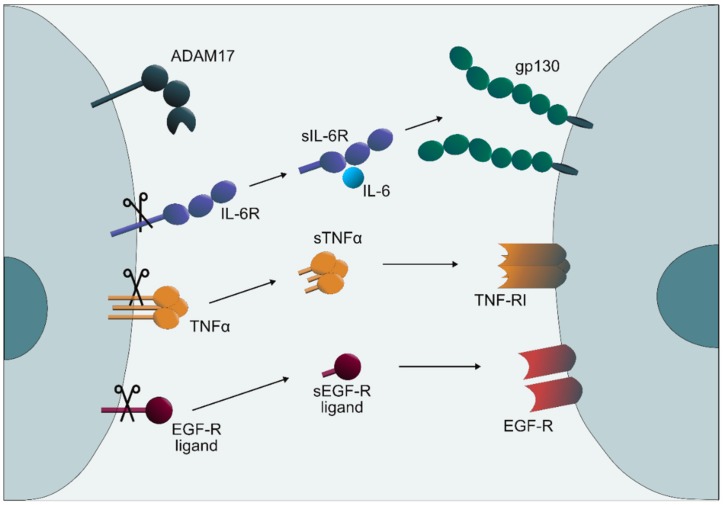
ADAM17 is a membrane-bound metalloprotease, which cleaves more than 80 substrates [44,45]. Major substrates of ADAM17 are IL-6R, TNFα, and ligands of the EGF-R. Cleavage of the IL-6R is a prerequisite of the pro-inflammatory IL-6 trans-signaling pathway via the sIL-6R. Cleavage of TNFα and the formation of soluble TNFα (sTNFα) leads to the stimulation of the pro-inflammatory TNFα receptor 1 (TNF-R_I_). Cleavage of ligands of the EGF-R is needed for the systemic activity of the ligands and therefore for the full stimulation of the EGF-R [47].

Since ADAM17 orchestrates at least three major signaling pathways—namely TNFα, IL-6R, and EGF-R signaling—it is not surprising that the regulation of protease activity is under tight control. Moreover, the involvement of ADAM17 in fundamental pathophysiologic processes such as inflammation and cancer can be envisaged. Of note, the importance of ADAM17 has been largely overlooked by all transcriptomics based studies since the mRNA expression of ADAM17 does not show major changes between organs as well as in health and disease [44,45]. The role of ADAM17 and IL-6 trans-signaling in inflammation and cancer will therefore be reviewed in the following paragraphs (Figure 2).

## 2. Results

### 2.1. IL-6 Trans-Signaling and ADAM17 in Inflammation

IL-6 is essential for the induction, progression and resolution of local and systemic inflammatory responses that activate acute phase reaction, promote tissue damage and development of autoimmune reactions, and plays a pivotal role in transition from innate to adaptive immune responses [55].

IL-6 knock-out mice are protected from development of experimental autoimmune encephalomyelitis (EAE), the mouse model of human multiple sclerosis [56,57,58] and mouse models of rheumatoid arthritis [59,60]. These mice also fail to efficiently control bacterial and viral infections with vaccinia virus, *Listeria monocytogenes* and *Mycobacterium tuberculosis* [31,61]. Similar to IL-6-deficient mice, IL-6R knock-out mice display a deficit in acute phase response, compromised wound healing, and reduced cellular infiltration during inflammation [62]. While acute phase reactions and defense against bacteria is mediated via membrane-bound IL-6R [63] delayed macrophage invasion in mouse models of inflammation was dependent on the availability of sIL-6R [64,65,66].

The cellular origin of sIL-6R was longtime unknown. Since it is evident that increased sIL-6R levels correlate with infiltrated leukocytes in various inflammatory pathologies like arthritic joints [67] and acute inflammation [64] macrophages were considered as a main source of sIL-6R. Furthermore, IL-6 stimulates polarization and proliferation of M2 macrophages via induction of IL-4R expression as shown in mouse models of obesity [68,69].

T cell responses can also be governed by both forms of IL-6 activities. Whereas T_reg_ development during experimental airway inflammation is controlled via membrane-bound IL-6R [70], IL-6 trans-signaling orchestrates T cell recruitment in an experimental peritoneal inflammation model [71]. Recently, it was found that TGFß, together with IL-6, drives the initial differentiation from naïve T-cells to pathogenic IL-17-producing T-cells (T_H_17 cells), which are key factors for induction of tissue damage in a variety of chronic inflammatory and autoimmune diseases [72,73]. Due to its complex role in immune modulation, it is essential to understand how IL-6 contributes to the respective pathology.

#### 2.1.1. Rheumatoid Arthritis

The first successful biologics-based therapy of rheumatoid arthritis patients was developed in the early 1990s by Marc Feldman and Ravinder Maini at the Charing Cross Hospital, London, UK and made use of antibodies neutralizing TNFα [74]. These TNFα antibodies had originally been prepared for clinical trials in human sepsis, which, however, entirely failed [75,76]. The first clinical trial with TNFα antibodies, which only involved few rheumatoid arthritis patients, was highly successful and led finally to the development of several TNFα-blocking drugs, which by now changed the perception of rheumatoid arthritis from a vastly debilitating disease to a largely manageable condition [76,77].

A decade ago, blocking IL-6R signaling with anti-IL-6R antibodies was approved for treatment of rheumatoid arthritis [78]. Beforehand, mouse models of rheumatoid arthritis had shown that IL-6 signaling drives disease progression by stimulating synovial hyperplasia, preservation of joint inflammation, and damage of underlying cartilage and bone [79,80,81]. All these disease symptoms are largely regulated by STAT3. Interestingly, it turned out that monotherapy with anti-IL-6R antibodies was superior to treatment of patients with the TNFα antibody adalimumab [82,83].

Mice which carry a Y757F mutation in the cytoplasmic tail of gp130 are unable to initiate the SHP2–MAP kinase–PI3 kinase axis because Y757 of gp130 is the docking site of SHP2 [84,85]. These mice can still activate the STAT1/STAT3 pathway [86,87]. Furthermore, these mice do not show negative regulation by SOCS3 since also this protein requires phosphorylated Y757 for its negative feedback activity [88,89]. These so-called gp130^F/F^ mice therefore show increased STAT1/STAT3, but no SHP2–MAP kinase–PI3 kinase signaling [86]. Besides enhanced autoantibody production against DNA, amplified cell infiltration into the joints and enhanced osteoclast activation, gp130^F/F^ mice develop arthritis-like symptoms within one year of age, thus resembling human rheumatoid arthritis [87]. Furthermore, clonal deletion of activated T cells was altered and these chronically activated T cells showed persistent IL-6-induced STAT3 and JAK1 phosphorylation indicating that IL-6 signaling in T cells plays a critical role in disease progression of rheumatoid arthritis [87]. In contrast, a dominant-negative STAT3 mutant with reduced STAT3 activity exhibits diminished cell infiltration into the joints, pannus formation, and cartilage damage in experimental arthritis in mice [90].

IL-6 signaling initiates a range of degenerative and inflammatory processes during rheumatoid arthritis. In vitro experiments suggested a possible effect of IL-6 and its soluble receptor on synovial fibroblasts proliferation [91] and survival [92]. Furthermore, IL-6 trans-signaling promotes the generation of osteoclasts [93,94,95] and bone resorption by stimulating the expression of receptor activator of nuclear factor kappa κ B (NFκB) (RANK) ligand (RANKL), which stimulates RANK expressed on osteoclast precursors leading to differentiation to bone-resorbing osteoclasts [96]. Binding of RANKL to RANK activates downstream signaling pathways like NFκB, MAPK, JNK, and Akt, which consequently stimulate the expression of osteoclastic transcription factors in osteoclast precursors. The study of Feng et al. [97] showed that IL-6 trans-signaling attenuated RANKL-induced ERK and JNK activation suggesting that IL-6 trans-signaling differentially regulates RANKL-induced osteoclastogenesis.

Enhanced proliferation and activation of synovial fibroblasts and osteoclasts leads to irreversible destruction of cartilage and bone of the affected joint. The majority of these processes could be abrogated by blocking IL-6 trans signaling, which coordinated leukocyte infiltration and severity of joint damage [79,98,99].

Interestingly, EGF-R inhibition reduced the severity of experimental arthritis, most likely due to reduction of synovial fibroblasts proliferation, cytokine production, and reduction of osteoclastogenesis [100]. Furthermore, iRhom2-dependent trafficking of the IL-6R sheddase ADAM17 and its activity on myeloid cells was shown to be involved in development of experimental arthritis in mice [101]. Since ADAM17 mediates the release of TNFα, sIL-6R, and EGF-R ligands, its activity is likely to play an important role in the development of rheumatoid arthritis (see below).

#### 2.1.2. Acute Inflammation

Rapid infiltration of neutrophils followed by replacement of mononuclear leukocytes is crucial for proper resolution of an acute inflammatory situation. IL-6 signaling via its soluble receptor regulates expression of CXC and CC chemokines that are needed for the switch of initial neutrophil influx to attraction of monocytes. In this context, IL-6 controls neutrophil infiltration by suppressing neutrophil-attracting chemokines and directs their apoptosis [64,102]. These effects were shown to be dependent on STAT3 activity [103]. The switch from neutrophil to monocytic cells could be abrogated by blocking IL-6 trans-signaling with the sgp130Fc protein in an acute peritonitis model [64,104] and in an air pouch model of acute inflammation in mice [65]. Furthermore, it was described that depletion of infiltrating neutrophils reduced the amount of sIL-6R in a model of local and acute inflammation. Thus, it was reasoned that in local and acute inflammation processes, neutrophils were a major source of sIL-6R [54]. This led us to investigate if proteolysis by ADAM17 is needed for the local release of sIL-6R and resolution of an acute inflammation. Contrary to our expectations, local ADAM17 activity was not critical for proper cell infiltration in acute inflammation in mice since the sIL-6R was infiltrating from the circulation in an ADAM17-independent process. These studies, however, confirmed the critical role of IL-6 trans-signaling in local and acute inflammation [105].

Initial recruitment of neutrophils and IL-6 secretion during acute peritonitis was described to be dependent on IFNγ. Defective IL-6 secretion into the site of inflammation in IFNγ^−/−^ mice could be restored by treatment with recombinant IFNγ and IL-1ß, but not with IFNγ alone [102]. IL-6 trans-signaling was furthermore shown to be critical for transition between innate and acquired immune responses. T cell recruitment was impaired in IL-6^−/−^ mice in a mouse model of peritonitis due to reduced chemokine expression which was mediated by STAT3 activation [71].

Unresolved inflammation leads inevitably to chronic fibrotic tissue damage. IL-6^−/−^ mice were protected from the development of fibrosis in recurrent inflammatory activation of peritonitis. In this particular model, IL-6 was needed for survival and expansion of Th1 cells, which were shown to be crucial for STAT1 activation in stromal cells [106]. Recently, it was shown that IL-6-mediated STAT1 phosphorylation, which reflects the effector characteristics of CD4^+^ T cells, is critically regulated by the tyrosine phosphatases PTPN2 and PTPN22. These two phosphatases were described to limit STAT1-mediated IL-6 signaling in both activated and memory CD4^+^ T cells thus modulating the expression of genes associated with particular immune responses [107]. 

#### 2.1.3. Sepsis

Mice with gp130-dependent STAT3 hyperactivation (gp130^F/F^) were hypersensitive to LPS-induced endotoxic shock. Overall survival of these mice was reduced accompanied by increased IL-6 expression and neutrophil influx into the peritoneal cavity. Both, genetic ablation of IL-6 in gp130^F/F^ or wildtype mice and specific inhibition of IL-6 trans-signaling led to an increased overall survival of LPS-induced endotoxic shock, suggesting that IL-6 signaling via the sIL-6R triggers the proinflammatory actions of IL-6 during LPS-induced endotoxic shock [108].

In a large animal study with 120 mice, the effect of total IL-6 blockade or selective inhibition of IL-6 trans-signaling on survival of sepsis induced by cecal ligation puncture was compared. Interestingly, inhibition of IL-6 trans-signaling via sgp130Fc was far superior to global IL-6 signaling blockade with an IL-6 neutralizing antibody. In this model, mice treated with sgp130Fc showed an up to 100% survival rate, an intact acute phase response, and less intestinal epithelial cell apoptosis, whereas no benefit was seen upon global IL-6 signaling blockade [109]. This enhanced therapeutic efficacy of inhibiting only the IL-6 trans-signaling pathway might be due to regenerative effects of IL-6 via the classic signaling pathway that was left intact when only IL-6 trans-signaling was blocked [12,110]. 

Deficiency of ADAM17 in inflammatory mouse models was shown to greatly diminish inflammatory responses [46,111,112]. When myeloid cells lacked ADAM17, mice showed slightly better survival rate after cecal ligation puncture. Blood and peritoneal bacteria levels, as well as inflammatory cytokine and chemokine plasma concentrations, were reduced in myeloid ADAM17-deficient mice, while increased neutrophil influx was observed [113]. This was possibly due to reduced proteolysis of the chemokine receptor CXCR2 or L-selectin [114,115].

#### 2.1.4. Inflammatory Bowel Disease

IL-6 has been described as a T cell activation factor and shown to induce T cell proliferation and survival [116,117]. This ability has been linked to the pathology of several models of chronic inflammatory disease [87,118]. In several animal models of intestinal inflammation, the disease could be alleviated by specific blockade of IL-6 trans-signaling with sgp130Fc. Blockade of IL-6 trans-signaling led to beneficial T-cell apoptosis in the colon of mice with chronic intestinal inflammation [118], since an enhanced T-cell resistance against apoptosis may contribute to disease perpetuation in Crohn’s disease [119]. In a murine model of intestinal inflammation using SAMP1/Yit mice, which is considered to be similar to inflammatory bowel disease in humans, treatment with sgp130Fc improved inflammation, as validated by less destruction of the epithelial layer and by less inflammatory infiltrates in the lamina propria of the small intestine [120]. In patients with inflammatory bowel disease, treatment with the IL-6R neutralizing antibody Tocilizumab suggested a clinical benefit [121], but this treatment has not been approved by the FDA. It is possible that, like in the murine cecal ligation puncture model of sepsis, specific blockade of IL-6 trans-signaling with sgp130Fc might be superior to global IL-6 blockade due to regenerative effects of IL-6 via the classic signaling pathway [12,110]. Of note, the sgp130Fc protein under the WHO name Olamkicept is currently tested in phase II clinical trials in patients with inflammatory bowel disease [20].

#### 2.1.5. Lung Pathophysiology: Emphysema, Asthma, and Idiopathic Pulmonary Fibrosis

Proinflammatory actions of IL-6 are implicated in the pathogenesis of lung emphysema and patients with COPD [122]. Analysis in mice revealed that development of emphysema was reliant on IL-6. In contrast to wt mice, gp130^F/F^ mice showed spontaneous development of lung emphysema at 6 months of age due to alveolar cell apoptosis, which was abrogated by genetic deletion of IL-6. Treatment of wt mice with cigarette smoke increased emphysema-associated lung volume increase, which was not seen in IL-6-deficient animals [123]. In both models, in the spontaneous development of lung emphysema seen in gp130^F/F^ mice and in cigarette smoke treated wildtype mice, treatment with sgp130Fc abrogated alveolar cell apoptosis and disease pathogenesis. This study furthermore revealed a possible connection of IL-6 trans-signaling and the mTOR pathway. After blockade of the serine/threonine kinase mTOR with rapamycin, IL-6 mediated lung pathologies were significantly reduced [124].

IL-6 was claimed to be important for the maintenance of the balance of effector T cells and regulatory T cells in lung pathogenesis. In a mouse model of asthma, IL-6 trans-signaling rather supported Th2 cytokine production, whereas classic IL-6 signaling was needed for the development of FoxP3^+^ regulatory T cells in the lung [70]. A recent study revealed an IL-6 trans-signaling specific gene signature in patients with asthma, which was accompanied by an increase in submucosal T cell and macrophage infiltration. Stimulation of primary human bronchial epithelial with both IL-6 and sIL-6R induced genes associated with airway remodeling such as matrix metalloproteinases and reduced expression of genes associated with epithelial barrier function such as ß-catenin. The gene signature found in this study was hypothesized to be helpful in determining patient subsets with high submucosal-inflammation and poor asthma control [125].

Idiopathic pulmonary fibrosis (IPF) characterizes a heterogeneous, chronic, and irreversible disorder in which deposition of collagen results in the loss of lung function. Although it is unsure if pulmonary fibrosis is the result of an unresolved inflammatory process the role of inflammatory cytokines is discussed. A study of cytokine levels in serum of patients with IPF revealed that IL-6 and IL-8 were increased in individuals with exacerbated disease state and that these increased levels correlated with a worse outcome [126]. This deleterious impact of IL-6 on the development of lung fibrosis was analyzed in different mouse models. In adenosine deaminase-deficient mice, which develop pulmonary inflammation and remodeling as well as in bleomycin-induced lung injury, IL-6 was shown to drive cellular infiltration and collagen deposition in the lungs [127,128]. A role for IL-6 trans-signaling in IPF was suggested as elevated sIL-6R levels were found in the lungs of patients with IPF and of mice with bleomycin-induced pulmonary fibrosis. Neutralization of IL-6 trans-signaling with sgp130Fc led to reduction of pulmonary inflammation, diminished myofibroblast accumulation and improved respiratory functions [129]. The development of pulmonary fibrosis in mice and differentiation of lung fibroblasts to collagen-producing myofibroblasts involves STAT3 activation by TGFß or IL-6 trans-signaling [130]. It was suggested that increased STAT3 activity in fibroblasts from IPF patients led to reduced apoptosis and thus contributes to the persistence of these cells [131,132].

### 2.2. IL-6 Trans-Signaling and ADAM17 in Cancer

It is widely accepted that inflammatory cytokine signaling plays key roles at many stages of tumorigenesis and supports malignant cell proliferation and metastasis [133]. Intestinal inflammation in several different animal models was efficiently blocked by a blockade of IL-6 trans-signaling [118,120] using sgp130Fc. On the other hand, intestinal inflammation was exacerbated by the application of Hyper-IL-6, a fusion protein of IL-6 and sIL-6R, which is a strong stimulator of IL-6 trans-signaling [134]. Therefore, it was reasonable to assume that inflammation associated cancer might also be driven by IL-6 trans-signaling [135].

#### 2.2.1. Colorectal Cancer

When mice were treated with a low dose of the mutagen azoxymethane (AOM) followed by consecutive cycles of orally administered dextran sulfate sodium (DSS), they developed small visible lesions around day 20, followed by the appearance of large tumors until day 80 [136]. When analyzing RAG-1 knockout mice, which are defective in B-cell and T-cell receptor recombination and therefore lack B- and T-lymphocytes and hence are severely immune-deficient, it became clear that these animals did not develop large tumors indicating that lymphocytes are involved in the regulation of tumor growth [136]. Colon samples from different time points after AOM/DSS treatment demonstrated massive IL-6 and sIL-6R accumulation from day 21 onwards. Treatment of the mice with a neutralizing antibody against IL-6R drastically reduced the tumor score [136]. Interestingly, also selective blockade of IL-6 trans-signaling by injection of the sgp130Fc protein effectively suppressed colon carcinogenesis, suggesting that IL-6 trans-signaling by inducing STAT3 phosphorylation and expression of the anti-apoptotic proteins bcl-xl and bcl-2 was responsible for growth of epithelial tumor cells [136]. In human colon tumor tissues, it was shown that the expression and activity of ADAM17 was significantly increased as compared with normal tissue, underlining an important role of ADAM17 in mediating the IL-6 trans-signaling response via the sIL-6R [137]. In a different intestinal tumor model, mice repeatedly treated only with 9 DSS but not with AOM developed intestinal tumors after 18 weeks. The incidence and number of tumors was drastically reduced when the mice were treated with the sgp130Fc protein. Interestingly, it was shown that lamina propria macrophages via induction of ADAM17 were important for the generation of sIL-6R and therefore for the induction of IL-6 trans-signaling [138].

When IL-6^−/−^ mice were compared to wt mice in their susceptibility to inflammatory colon cancer in the AOM/DSS model, it turned out that IL-6^−/−^ mice had less tumors but more inflammation in the intestine, arguing for a role of IL-6 in the intestinal regeneration response. [110]. Indeed, it was shown in the same study that deletion of STAT3 in the intestinal epithelium led to more severe DSS colitis with pronounced colonic ulcerations and body weight loss, indicating that the IL-6 response in the intestinal epithelial cells was important for regeneration [110]. These data were further corroborated by a parallel study in which the authors used transgenic mice expressing in intestinal epithelial cells a constitutively active form of gp130 dimerized by a leucine zipper [6], which led to cell-autonomous STAT3 activation. These mice were largely resistant to DSS-induced colitis [139]. The above data suggested a role of IL-6 in the regeneration of the intestine. Indeed, treatment of mice with recombinant IL-6 protected the animals from DSS-induced colitis whereas treatment with a neutralizing IL-6 antibody aggravated DSS-induced colitis [140,141].

Inflammatory hepatocellular adenomas often harbor mutations in the gp130 gene, which leads to ligand independent activation of gp130 [142]. Transgenic mice, which express constitutively active gp130 transgene developed aberrant proliferation of intestinal epithelial cells. Interestingly, it was observed that the YAP and Notch signaling pathway were activated by the constitutively active gp130 mutant [5]. Furthermore, it was reported by the same authors that loss of APC in mice and in human patients led to the activation of Src, YAP, Notch, and STAT3. The presence of the activated gp130 transgene in intestinal epithelial cells accelerated the development of colorectal cancer and depended on the activities of Src family and JAK kinases [4].

**Figure 3:**
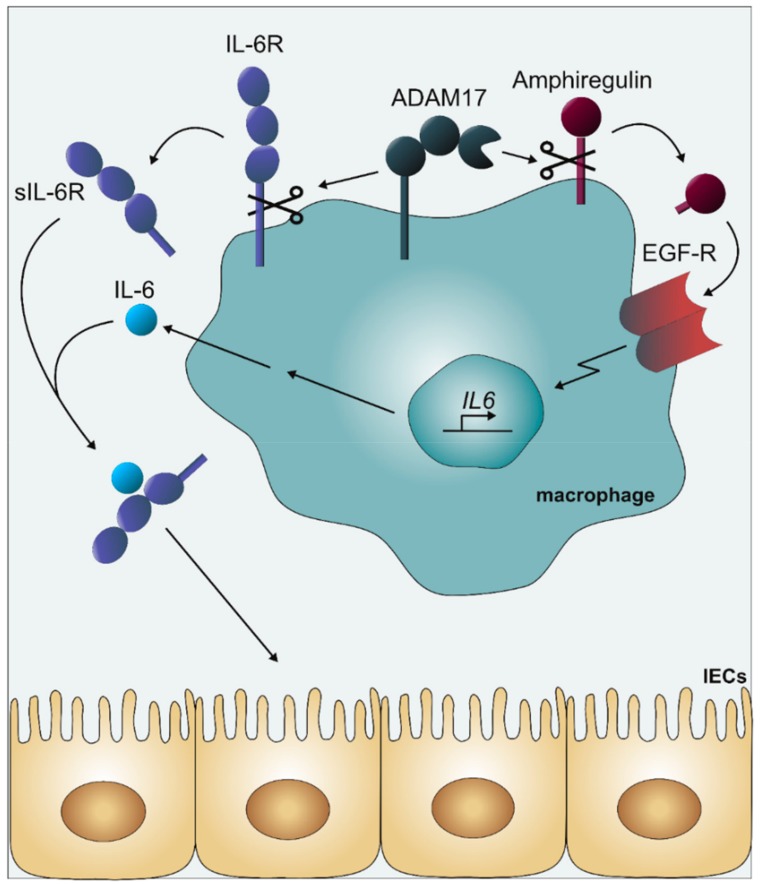
Involvement of ADAM17 in the formation of colon cancer. Under inflammatory conditions, ADAM17 cleaves the membrane-bound EGF-R ligand amphiregulin, leading to the stimulation of the EGF-R on macrophages. This EGF-R stimulation on macrophages leads to increased IL-6 synthesis via transcriptional stimulation. Moreover, activation of ADAM17 under inflammatory conditions leads to cleavage of the membrane-bound IL-6R on macrophages to generate soluble IL-6R (sIL-6R). IL-6 in complex with sIL-6R leads to stimulation of intestinal epithelial cells (IECs) via IL-6 trans-signaling resulting in colon cancer formation [144].

Mice with a complete deletion of the ADAM17 gene are not viable [42]. Although, ADAM17 floxed mice are available [46], it was not completely clear in which cell types we wanted to delete the ADAM17 in order to show a decisive role of the protease, since many cells including macrophages can be the source of the sIL-6R in vivo [2]. We therefore made use of ADAM17 hypomorphic ADAM17^ex/ex^ mice, which we had engineered to only express about 5% of ADAM17 protein levels in all cells [47]. ADAM17 hypomorphic ADAM17^ex/ex^ mice were shown to be hypersensitive to DSS-induced colitis due to a failure to induce EGF-R signaling, which was needed for intestinal regeneration. EGF-R activation could be compensated by treatment of the mice with recombinant ligands of the EGF-R, which compensated for the lack of cleavage of these membrane-bound proteins [47]. We therefore applied a colon cancer model to ADAM17^ex/ex^ mice. In APC^min/+^ mice, a heterozygous germ line truncation in the APC gene results in the formation of intestinal tumors after spontaneous loss of heterozygosity of the remaining APC wt allele [143,144]. Strikingly, in the absence of ADAM17 activity, almost no intestinal tumors were detected and the few remaining tumors were only of low-grade dysplasia whereas in the presence of ADAM17, also high-grade dysplasias and invasive carcinomas were detected [144]. Surprisingly, we failed to detect significantly elevated levels of phosphorylated EGF-R on intestine sections of wt and ADAM17^ex/ex^ mice (Figure 3) [144].

Elevated activity of the EGF-R has been associated with an increased risk of colon carcinogenesis [144,145]. Recently, it was reported that in patients, EGF-R was expressed on myeloid cells within intestinal tumors and that in mice, EGF-R on myeloid cells but not on intestinal epithelial cells promoted colitis associated cancer in the AOM/DSS model and tumorigenesis in the APC^min/+^ model and protects from colitis in an independent fashion [141]. Moreover, EGF-R activation in myeloid cells led to strongly increased synthesis and secretion of IL-6 [141].

ADAM17 on myeloid cells can lead to the shedding of ligands of the EGF-R such as amphiregulin and to the shedding of the IL-6R [10]. We therefore speculated that IL-6 trans-signaling initiated by the EGF-R mediated IL-6 secretion and the ADAM17-mediated IL-6R shedding might contribute to colon cancer formation in the APC^min/+^ model. We therefore crossed APC^min/+^ mice with either IL-6^−/−^ mice or mice, which were transgenic for sgp130Fc and in which IL-6 trans-signaling was blocked [19,65]. Indeed, tumorigenesis was strongly reduced to the same extent in IL-6^−/−^ mice and sgp130Fc transgenic mice indicating that IL-6 trans-signaling strongly contributed to tumor formation in the APC^min/+^ model [144]. Furthermore, in a chemical colon cancer model, in which mice were repeatedly treated with AOM but not with DSS, sgp130Fc transgenic mice were protected from colon cancer [144]. We concluded from these data that in colon cancer, IL-6 trans-signaling apparently acted downstream of the EGF-R and blockade of IL-6 trans-signaling might represent a novel therapeutic window for patients resistant to anti-EGF-R antibodies (Figure 3) [146].

#### 2.2.2. Pancreatic Cancer

In the Kras^G12D^ pancreatic cancer model, strong non-cell-autonomous STAT3 activation in tumor cells was observed [147]. IL-6 mRNA levels were significantly increased in tumor tissue. The cellular source of IL-6 was infiltrating immune cells, mainly F4/80-positive macrophages. When the pancreatic Kras^G12D^ mice were crossed with IL-6^−/−^ mice or sgp130Fc transgenic mice. IL-6^−/−^ mice had fewer and predominantly low grade pancreatic intraepithelial neoplasias. Interestingly, blocking IL-6 trans-signaling had a similar effect as deletion of the IL-6 gene since sgp130Fc transgenic mice also showed strongly reduced tumor numbers. This decrease in tumor development was paralleled by a marked reduction of STAT3 phosphorylation [147]. It was speculated that the concept of non-cell-autonomous STAT3 activation of tumor cells brought about by IL-6 trans-signaling was a general phenomenon, which might also be applied to other neoplasias and cancer diseases [147].

#### 2.2.3. Liver Cancer

During liver regeneration, quiescent hepatocytes regain their ability to enter the cell cycle. Importantly, IL-6 [148] and in particular IL-6 trans-signaling has been found to be required for liver regeneration [149]. Accordingly, in IL-6/sIL-6R double transgenic mice, permanent hepatocyte proliferation and formation of adenomas was observed, indicating a possible role of IL-6 trans-signaling for hepatocellular neoplasia [150,151]. Treatment of mice with diethylnitrosamine is used as one of the standard models for liver cancer [152]. In this model, it has been recognized that IL-6 deficient mice develop substantially less tumors as compared to wt mice [153]. When sgp130Fc transgenic mice were treated with diethylnitrosamine, it turned out that myeloid cell derived sIL-6R increased upon treatment and liver tumor development the animals was largely blocked [154]. We concluded from these experiments that IL-6 trans-signaling but not IL-6 classic signaling was involved in the development of hepatocellular carcinoma, at least in the diethylnitrosamine model [154].

#### 2.2.4. Lung Cancer

As mentioned above, gp130 signal transduction is mediated by the SHP2–MAP kinase–PI3 kinase axis. In addition, STAT1 and STAT3 become recruited to the cytoplasmic tail of gp130, are phosphorylated by JAK1, and dimerize and travel to the nucleus to act as transcription factors [3]. Mice, which carry a Y757F mutation in the cytoplasmic tail of gp130 are unable to initiate the SHP2–MAP kinase–PI3 kinase axis because Y757 of gp130 is the docking site of SHP2. These mice can still activate the STAT1/STAT3 pathway [86]. Furthermore, these mice do not show negative regulation by SOCS3 since also this protein requires phosphorylated Y757 for its negative feedback activity [86]. These so-called gp130^F/F^ mice therefore show increased STAT1/STAT3 but no SHP2–MAP kinase–PI3 kinase signaling [86].

The gp130^F/F^ mouse model has been widely used to explore a range of gp130 mediated disease states. The more pronounced gp130/STAT1/STAT3 response seen in these mice has been used to analyze novel therapeutic strategies for inflammatory diseases. Gp130^F/F^ mice are more sensitive than wt mice towards endotoxin. It was found that genetic ablation of IL-6, antibody-mediated inhibition of the IL-6R or blockade of IL-6 trans-signaling by the sgp130Fc protein completely protected gp130^F/F^ mice from endotoxin hypersensitivity [108]. Gp130^F/F^ mice spontaneously develop lung emphysema, which could be prevented by blocking IL-6 trans-signaling [124]. When floxed Kras^G12D^ mice were treated via intranasal inhalation with adenovirus, which encoded cre recombinase 6 weeks prior to analysis, massive lung cancer development was observed, which was much more pronounced in gp130^F/F^ mice as compared to wt mice [155]. Ablation of IL-6 or STAT3 suppressed the extent of lung cancer in this model [155]. Furthermore, we could demonstrate that specific inhibition of IL-6 trans-signaling by the sgp130Fc protein significantly ameliorated lung cancer pathogenesis [155].

Since IL-6 trans-signaling depends on the proteolysis of the membrane-bound IL-6R by the metalloprotease ADAM17, we asked whether in the absence of ADAM17 activity, lung tumor formation in the Kras^G12D^ mouse model would be blocked. When ADAM17^ex/ex^ mice were crossed with Kras^G12D^ mice, tumor formation in the lung was largely inhibited [156]. Interestingly, the cytoplasmic portion of ADAM17 was phosphorylated in Kras^G12D^ mice but not in wt mice [156]. Phosphorylation of the cytoplasmic portion of ADAM17 has been shown to lead to activation of the enzyme [157]. Interestingly, in Kras^G12D^ mice, the levels of sIL-6R were significantly elevated, whereas in compound ADAM17^ex/ex^: Kras^G12D^ mice, sIL-6R levels were comparable to wt mice. No changes in soluble TNFα levels were observed [156]. Moreover, in a xenograft model with ADAM17-deleted A549 cells, tumor formation was abrogated as compared to unedited A549 cells [156]. Along the same line, pharmacologic inhibition of ADAM17 activity also led to decreased tumor formation in Kras^G12D^ mice together with decreased sIL-6R levels [156]. Moreover, in a nicotine-derived nitrosamine ketone model of tobacco-related lung cancer, genetic or pharmacologic blockade of ADAM17 led to a significant reduction of lung lesions and concomitant reduced sIL-6R levels [158]. Together, these data indicated that ADAM17 via IL-6 trans-signaling was a prerequisite of lung tumor formation in these animal models.

## 3. Conclusions and Outlook

ADAM17 activation orchestrates three major signal transduction pathways, namely IL-6 trans-signaling, stimulation of the TNFα signaling pathway via TNF-R_I_ and the EGF-R pathway via cleavage of EGF-R ligands such as amphiregulin, TGFα, or Hb-EGF. Apparently, in many inflammatory diseases as well as in colon cancer and lung cancer, the IL-6 trans-signaling pathway plays a dominant role as compared to the TNFα signaling pathway (Table 1).

The dependence of IL-6 trans-signaling via the generation of IL-6 and sIL-6R by the EGF-R on macrophages demonstrates the existence of a possible novel therapeutic window for the treatment of colon cancer and lung cancer, which might be important in view of the fact that EGF-R antibodies including cetuximab or pantuximab are only useful in patients in whom there are no activating KRAS mutations [161,162]. Even in such patients without KRAS mutations, who show an initial response, almost invariably resistance against EGF-R blockade is seen [163]. In all of these cases, blockade of the IL-6 trans-signaling pathway might offer an additional and alternative treatment option. This treatment option, however, will need to be verified in future human studies.

## Figures and Tables

**Table 1:** Efficacy of IL-6 trans-signaling blockade by sgp130Fc in preclinical models of inflammation and inflammation associated cancer.

Disease Model	Outcome of the Study
Intestinal inflammation [118,138]	Suppression of colitis activity
Acute inflammation [54,64,65]	Blockade of inflammatory processes
Atherosclerosis [159]	Regression of advanced atherosclerosis
Rheumatoid arthritis [79,98,99]	Improvement of established arthritis
Sepsis [108,109]	Up to 100% survival in different sepsis models
Pancreatitis-lung failure [160]	100% survival of severe acute pancreatitis
Lung emphysema [124]	Improvement by blockade of alveolar cell apoptosis
Abdominal aortic aneurism [30]	Improved survival in two animal models
Colon cancer [110,136,137,138,144]	Blockade of tumor formation
Pancreatic cancer [147]	Inhibition of pancreatic neoplasia progression
Liver cancer [154]	Protection from tumor formation
Lung cancer [156,158]	Amelioration of lung cancer pathogenesis

## Abbreviations

ADAM	a disintegrin and metalloprotease
AOM	azoxymethane
APC	adenomatous polyposis coli protein
COPD	chronic obstructive pulmonary disease
DSS	dextransulfate sodium
EAE	experimental autoimmune encephalomyelitis
EGF	epidermal growth factor
FDA	US food and drug administration
FoxP3	forkhead box protein 3
FRMD	FERM domain containing protein, identical to iTAP
Gp130	glycoprotein 130 kDa
IECs	intestinal epithelial cells
IFN	interferon
IL-6	Interleukin-6
IL-6R	Interleukin-6 receptor
iRhom	inactive rhomboid protease
iTAP	iRhom Tail-Associated Protein identical to FRMD8
JAK	Janus kinase
JNK	c-Jun N-terminal kinase
Kras	oncogene first identified in Kirsten RAt Sarcoma virus
MAP	mitogen-activated protein
mTOR	mechanistic target of rapamycin
NFκB	nuclear factor kappa-light-chain-enhancer
Notch	cell signaling receptor present in most animals
PTPN	tyrosine phosphatase
RANK	Receptor Activator of NFκB
RANKL	ligand of RANK
ROSA26	reverse oriented splice acceptor, Clone 26
SHP2	protein-tyrosine phosphatase shp2
SOCS	suppressor of cytokine signaling
Src	proto-oncogene tyrosine-protein kinase
STAT	signal transducer and activator of transcription
TNF	tumor necrosis factor
WHO	world health organization
YAP	proto-oncogene yes associated protein.
